# Comparison of the 7^th^ and proposed 8^th^ editions of the AJCC/UICC TNM staging system for non-small cell lung cancer undergoing radical surgery

**DOI:** 10.1038/srep33587

**Published:** 2016-09-19

**Authors:** Ying Jin, Ming Chen, Xinmin Yu

**Affiliations:** 1 Department of Medical Oncology, Zhejiang Cancer Hospital, Hangzhou, China; 2 Zhejiang Key Laboratory of Radiation Oncology, Hangzhou, China; 3 Department of Radiation Oncology, Zhejiang Cancer Hospital, Hangzhou, China; 4 Zhejiang Key Laboratory of Diagnosis and Treatment Technology of Thoracic Oncology, Hangzhou, China.

## Abstract

The present study aims to compare the 7^th^ and the proposed 8^th^ edition of the AJCC/UICC TNM staging system for NSCLC in a cohort of patients from a single institution. A total of 408 patients with NSCLC who underwent radical surgery were analyzed retrospectively. Survivals were analyzed using the Kaplan –Meier method and were compared using the log-rank test. Multivariate analysis was performed by the Cox proportional hazard model. The Akaike information criterion (AIC) and C-index were applied to compare the two prognostic systems with different numbers of stages. The 7^th^ AJCC T categories, the proposed 8^th^ AJCC T categories, N categories, visceral pleural invasion, and vessel invasion were found to have statistically significant associations with disease-free survival (DFS) on univariate analysis. In the 7^th^ edition staging system as well as in the proposed 8^th^ edition, T categories, N categories, and pleural invasion were independent factors for DFS on multivariate analysis. The AIC value was smaller for the 8^th^ edition compared to the 7^th^ edition staging system. The C-index value was larger for the 8^th^ edition compared to the 7^th^ edition staging system. Based on the data from our single center, the proposed 8^th^ AJCC T classification seems to be superior to the 7^th^ AJCC T classification in terms of DFS for patients with NSCLC underwent radical surgery.

Despite screening and treatment progress, Lung cancer remains the leading cause of cancer death in the People’s Republic of China as well as worldwide. In 2015, an estimated 733,300 new cases of lung and bronchial cancer will be diagnosed, and 610,200 deaths are estimated to occur because of the disease^1^. Studies have shown, the overall 5-year relative survival rate for all lung cancer patients was less than 20%. On contrast, those patients with operable pulmonary tumors experienced more favorable outcomes that their 5-year survival rate ranged from 20% to 70%^2^.

Accurate evaluation of the tumor stage, including the extent of the pulmonary lesion and lymph node status, is essential for prognostic assessment and decision-making of the stage-specific therapeutic strategy. The American Joint Committee on Cancer (AJCC)/Union for International Cancer Control (UICC) tumor, node, metastasis (TNM) staging system is the main tumor-staging system used in clinical practice and research for various solid tumors, including lung cancer. Since the first edition published in 1977, every few years, the version of this classification system is updated according to new data^3^,^4^,^5^.

A new database of 77,156 patients is being used by the International Association for the Study of Lung Cancer (IASLC) now to inform the 8^th^ edition of the TNM classification of lung cancer due to be introduced in the near future, resulting in several changes from the 7^th^ edition, particularly as regards the T categories^6^. According to the proposed 8^th^ TNM edition, T categories were so redefined in order to improve their prognostic validity^7^: The T1 is subclassified into T1a (≤1 cm), T1b (>1 to ≤2 cm), and T1c (>2 to ≤3 cm); T2 is subclassified into T2a (>3 to ≤4 cm) and T2b (>4 to ≤5 cm); Tumors greater than 5 to less than or equal to 7 cm is reclassified as T3; Tumors greater than 7 cm is reclassified as T4.

It is unclear at present, whether these changes have significantly improved the prognostic ability. In this study, we aimed to investigate the predictive ability of the forthcoming 8^th^ edition of the AJCC/UICC TNM classification for disease free survival (DFS) and to compare this with the 7^th^ edition in a cohort of patients with NSCLC who underwent radical surgery with curative intent.

## Results

### Descriptive characteristics

A total of 408 patients were enrolled in this study. Patients’ characteristics are described in Table 1. All patients were of Chinese ethnicity with a male predominance (76.5%). The mean age of diagnosis of NSCLC was 59.9 years (ranging from 30 to 82 years). One hundred and seventy-seven (43.4%) patients received adjuvant chemotherapy (cisplatinum-based doublets) after operation, and among these patients, the mean cycle of chemotherapy is 3.50 (from 1 to 4).

When tumor size was the only consideration, according to the 7^th^ edition AJCC TNM stage, 58 patients (14.2%) were diagnosed as pathologic T1a (≤2 cm), 93 patients (22.8%) were T1b (>2 cm, ≤3 cm), 140 patients (34.3%) were T2a (>3 cm, ≤5 cm), 69 patients (16.9%) were T2b (>5 cm, ≤7 cm), 48 patients (11.8%) were T3 (>7 cm), and none of the patients were T4. According to the proposed 8^th^ edition AJCC TNM stage, 8 patients (2.0%) were diagnosed as pathologic T1a (≤1 cm), 50 patients (12.3%) were T1b (>1 cm, ≤2 cm), 93 patients (22.8%) were T1c (>2 cm, ≤3 cm), 93 patients (22.8%) were T2a (>3 cm, ≤4 cm), 47 patients (11.5%) were T2b (>4 cm, ≤5 cm), 69 patients (16.9%) were T3 (>5 cm, ≤7 cm), 48 patients (11.8%) were T4 (>7 cm).

In addition, in the 8 patients whose lesions <1 cm, the incidence of lymph node metastasis was zero; in the 50 patients whose lesions between 1 cm and 2 cm, the incidence of lymph node metastasis was 20%; in the 93 patients whose lesions between 2 cm and 3 cm, the incidence of lymph node metastasis was 37.6%; in the 93 patients whose lesions between 3 cm and 4 cm, the incidence of lymph node metastasis was 54.9%; in the 47 patients whose lesions between 4 cm and 5 cm, the incidence of lymph node metastasis was 48.9%; in the 69 patients whose lesions between 5 cm and 7 cm, the incidence of lymph node metastasis was 43.5%; in the 48 patients whose lesions >7 cm, the incidence of lymph node metastasis was 66.7%.

### Disease-free Survival

**Figure 1:**
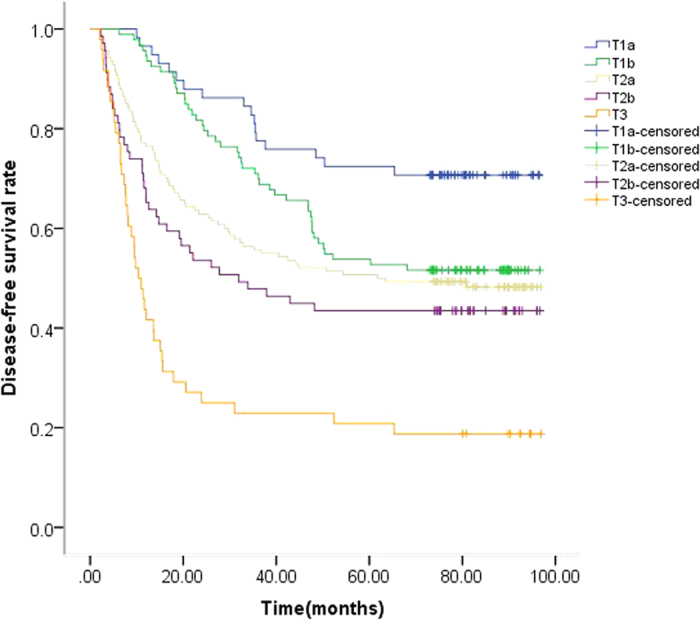
Kaplan-Meier estimates of disease-free survival (DFS) according to the 7^th^ edition T stage. The 5-year DFS rates were 72%, 54%, 51%, 43% and 21% for patients with T1a (≤2 cm), T1b (>2 cm, ≤3 cm), T2a (>3 cm, ≤5 cm), T2b (>5 cm, ≤7 cm), T3 (>7 cm), respectively. P < 0.001.

**Figure 2:**
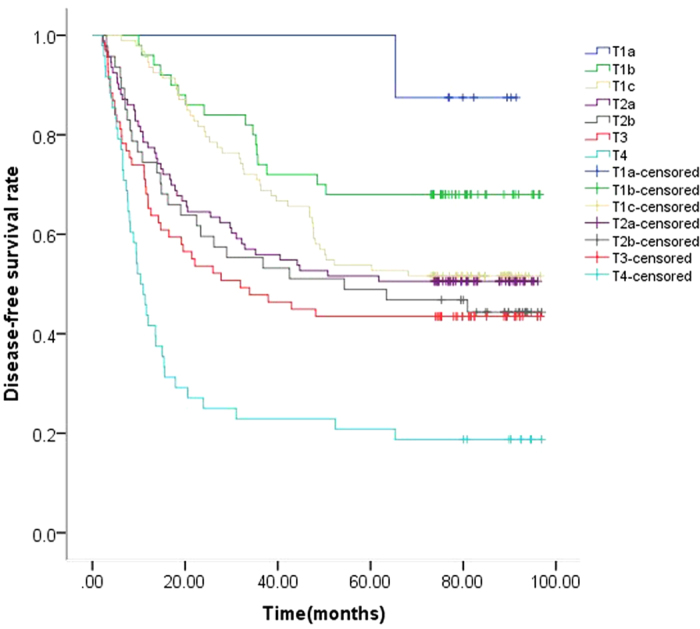
Kaplan-Meier estimates of disease-free survival (DFS) according to the proposed 8^th^ edition T stage. The 5-year DFS rates were 100%, 68%, 54%, 52%, 49%, 43% and 21% for patients with T1a (≤1 cm), T1b (>1 cm, ≤2 cm), T1c (>2 cm, ≤3 cm), T2a (>3 cm, ≤4 cm), T2b (>4 cm, ≤5 cm), T3 (>5 cm, ≤7 cm), T4 (>7 cm), respectively. P < 0.001.

Until the last follow-up checkpoint January 31, 2016, 212 patients (52%) were diagnosed with disease relapse or metastases. The median DFS was 52.4 months (ranging from 53.2 to 61.0 months). The 1-year, 2-year, 3-year DFS were 78%, 65%, 57%, respectively. As shown in Fig. 1, the five Kaplan-Meier survival curves did not overlapped each other in accordance with the 7^th^ AJCC system. The seven Kaplan-Meier survival curves did not overlapped each other in accordance with the proposed 8^th^ AJCC system (Fig. 2).

### Univariate analysis

Factors that were analyzed are listed in Table 1, the pathologic N stage (P < 0.001), visceral pleural invasion (P < 0.001), vessel invasion (P = 0.002), the pathologic 7^th^ T stage (P < 0.001) as well as the proposed pathologic 8^th^ T stage (P < 0.001) were significantly associated with DFS.

### Multivariate analysis

Since both the 7^th^ T stage and the proposed 8^th^ T stage were prognostic factors in the univariate analysis, two separate multivariate models were performed: one including pathologic N stage, visceral pleural invasion, vessel invasion, and the pathologic 7^th^ T stage; the other including pathologic N stage, visceral pleural invasion, vessel invasion, and the proposed pathologic 8^th^ T stage. As shown in Table 2, in the model in accordance with the 7^th^ edition, pathologic N stage (HR = 1.554), pleural invasion (HR = 1.395), and the pathologic 7^th^ T stage (HR = 1.330) were independent prognostic parameters for DFS. Also in the model in accordance with the 8^th^ edition, pathologic N stage (HR = 1.569), visceral pleural invasion (HR = 1.393), and the proposed pathologic 8^th^ T stage (HR = 1.230) were independent prognostic parameters for DFS. Then, the performance of the 7^th^ and the proposed 8^th^ systems were quantified by the likelihood ratio chi-square and AIC. The AIC value was smaller for the proposed 8^th^ edition compared to the 7^th^ edition AJCC TNM staging system, which indicates that the proposed 8^th^ edition has a better prognostic stratification. Then we performed the analysis of concordance index for the two models and the value for the proposed 8^th^ system was larger than the 7^th^ edition, which means it is more informative about patient’s outcome. The results are consistent with the AIC value.

## Discussion

The long-term survival of NSCLC after surgical resection is still unsatisfactory due to the high recurrence and metastasis^8^. Therefore, it is of great importance to identify prognostic factors which may help to stratify lung cancer patients after radical resection and select high-risk patients who should receive aggressive adjuvant treatment. The IASLC database includes large numbers of patients from several countries can serve as a powerful tool to explore the prognostic details about lung cancer.

The seventh edition of the AJCC TNM staging system for lung cancer has served for clinics since 2009. Based on extensive analyses and evidence from a new large international database^6^,^9^ (a database of 77,156 evaluable patients diagnosed with lung cancer from 1999 to 2010), the IASCLC made proposals^7^,^10^,^11^ to inform the 8^th^ edition of the TNM classification of lung cancer with intent to improve lung cancer staging system, allow for more accurate prediction of prognosis, and better guide lung cancer treatment options. The revision for the proposed 8^th^ edition compared to the 7^th^ edition of AJCC TNM staging system consisted of changes in the T descriptors that reclassifies tumor size into the more refined T subgroups, reclassify the classification of tumor involvement of main bronchus regardless of distance from carina, reclassify atelectasis/pneumonitis, reclassify diaphragm invasion and delete mediastinal pleural effusion as a T descriptor, and the subclassification of M1. Since the 7^th^ edition adequately predict the prognosis, the N descriptors remained the same in the forthcoming staging system.

In this paper, we compared the predictive ability of the forthcoming 8^th^ edition of the AJCC/UICC TNM classification for disease free survival (DFS) to the 7^th^ edition in 408 patients with NSCLC who underwent radical surgery with curative intent in our single center. The major finding of our investigation is that both the 7^th^ and proposed 8^th^ edition AJCC staging system identified the T descriptors as the independent prognostic factors for DFS. Although the AIC and C-index values were not obviously different between the two models, the proposed 8^th^ AJCC staging system T descriptors seems to be superior to that in the 7^th^ edition.

Tumor size is an important prognostic factor for long-term survival in Lung Cancer. Jeffrey PL *et al.*^12^ analyzed a cohort of patients with pathologically confirmed stage IA NSCLC and found that the 5-year survival for patients with tumor size ≤2.0 cm was higher than those with tumor size >2.0 cm (77.2% vs 60.3%, P = 0.03). In a review of 598 patients with stage I tumors, Martini *et al.*^13^ showed that the survival of patients with lesions <1 cm was significantly better than those with tumors >1 cm. In a recently published study, Zhang Yang *et al.*^14^ investigated 2,260 patients with N0M0 NSCLC in the Surveillance, Epidemiology and End Results (SEER) database and found that the 5-year OS rates of pathological tumor size ≤1 cm, 1–2 cm, 2–3 cm, 3–4 cm, 4–5 cm, 5–7 cm, and >7 cm were 77.8%, 74.1%, 68.2%, 64.5%, 58.7%, 53.2%, and 57.3%, respectively. In contrast to their result, our result revealed a more significant trend toward worse survival with increasing tumor size. In our analysis, 5-year DFS rates of pathological tumor size ≤1 cm, 1–2 cm, 2–3 cm, 3–4 cm, 4–5 cm, 5–7 cm, and >7 cm were 100%, 68%, 54%, 52%, 49%, 43%, and 21%, respectively. In our study, the 5-year DFS rate decreased from 100% in tumors ≤1 cm to 21% in tumors >7 cm, whereas in their study, the 5-year OS rate dropped from 77.8% to 57.3%. We think the most plausible explanation for this differences was that in their study the included patients with pathological N0M0 NSCLC, which may represent a group of patients with relative better prognosis compared with those with lymph nodes involvement, leading to selection bias and smaller differences in survival between patients with small and large tumors. In addition, in early-stage NSCLC, the DFS is more refined estimate of outcome than OS.

In our results, we did show that the incidence of lymph nodes involvement was higher in large tumors than small tumors: in the patients whose lesions <1 cm, the incidence of lymph node metastasis was zero; in those whose lesions between 1 cm and 2 cm, the incidence was 20%; in those whose lesions between 2 cm and 3 cm, the incidence was 37.6%; in those whose lesions >7 cm, the incidence was 66.7%. Previous researches reported similar results with ours. Ishida *et al.*^15^ reported that the incidence of lymph node metastasis in patients with lesions >2 cm was 38%, for lesions between 1 cm and 2 cm was 17%, and zero in lesions <1 cm. Our result provide further support for the theory that tumor size may reflect malignant behavior and that small lesions do represent early stage disease. It provides some reassurance that it is necessary to further subdivided the T classification as the proposed 8^th^ edition AJCC staging system.

The multivariate analysis also identified visceral pleural invasion and N classification as the independent prognostic factors for DFS. Several studies demonstrated such result. Wu CY *et al.*^16^ has established a predictive survival model for survival in patients with NSCLC who received radical resection and identified tumor size, visceral pleural invasion, and patients with lymph node metastasis as independent prognostic factor for DFS. Huang H *et al.*^17^ reported that visceral pleural invasion is a size-independent poor prognostic factor in stage I NSCLC patients. In a systemic review and meta-analysis, Jiang L *et al.*^18^ showed that visceral pleural invasion together with tumor size has a synergistic effect on survival in node-negative NSCLC. Indeed, the national comprehensive cancer network (NCCN) guideline recommend stage IB NSCLC patients with visceral pleural invasion to consider adjuvant chemotherapy after surgical resection.

There were several limitations in this paper. Firstly, we only analyzed patients with radical surgery, which may not completely reflect the advantages of the 8^th^ edition of the stage system. Secondly, the sample was relative small, and the differences of AIC and C-index between the two models were not obvious. Therefore, in the next study we will gather more data, in the hope that this will allow clearly distinguishing between the two models.

In conclusion, Based on the data from our single center, the proposed 8^th^ AJCC T classification seems to be superior to the 7^th^ AJCC T classification in terms of DFS for patients underwent radical surgery. Further studies with larger number of patients are needed in order to validate the generalizability of the proposed 8^th^ edition AJCC/UICC TNM staging system of lung cancer.

## Methods

### **P**atients Enrollment

Included were patients who underwent radical operation with curative intent from January 1, 2008 to December 31, 2009 at Zhejiang Cancer Hospital, Hangzhou, China for squamous cell carcinoma and adenocarcinoma of the lung. Study protocols were approved by the Ethical Review Community of Zhejiang Cancer Hospital. The requirement of informed consent was waived by the committee as it was a retrospective research. The methods were carried out in accordance with the relevant guidelines. Excluded were patients who had received neoadjuvant chemo (radio) therapy, patients who had received a non-curative (R1/R2) resection, patients who died in one month after surgery, and patients with other types of malignancy. Patients’ data were collected including the following variables: sex, age, histologic subtype, pathologic tumor size, pathologic lymph node involvement status, differentiation grade, visceral pleural invasion, and vessel invasion. The same patient dataset was used stage patients according to both the 7^th^ and 8^th^ edition system.

### Follow up procedures

Postoperatively, patients were followed at regular intervals by chest CT. DFS was defined as the time from the date of surgery to the time of relapse or metastases. Patients who did not relapse or metastases were censored on the day of last follow-up. The last follow-up checkpoint was January 31, 2016.

### Statistical Analysis

Statistical analysis was performed using SPSS22.0 package. Statistical significance was defined as when *P* < 0.05. Survivals were analyzed using the Kaplan –Meier method and were compared using the log-rank test. Multivariate analysis was performed by the Cox proportional hazard model. To measure homogeneity of the direct comparison of the two different edition stage systems, the likelihood ratio χ^2^ test related to the Cox regression model was used. The discriminatory ability and monotonicity of gradient assessments were measured with the linear trend χ^2^ test of survival curves according to the N classification of the 6^th^ and 7^th^ editions. The Akaike information criterion (AIC) as well as the C-index were applied into the Cox proportional hazard regression model to correct for the potential bias in comparing prognostic systems with different numbers of stages. AIC was defined as follows: AIC = −2 log maximum likelihood +2 × (the number of parameters in the model). A smaller AIC value indicated a better model for predicting outcome^19^. C-index were performed using R software (version 3.2.4), and larger C-index value indicated a better model for predicting outcome.

## Additional Information

**How to cite this article**: Jin, Y. *et al.* Comparison of the 7^th^ and proposed 8^th^ editions of the AJCC/UICC TNM staging system for non-small cell lung cancer undergoing radical surgery. *Sci. Rep.* **6**, 33587; doi: 10.1038/srep33587 (2016).

## Figures and Tables

**Table 1:** Patient demographics and results of univariate analysis for disease-free survival.

Total N = 408	Value	5-y survival, %	*Log-rank X*^2^ *value*	*P*
Age			0.080	0.777
≥60	224 (45.1)	49		
<60	184 (54.9)	51		
Gender			2.059	0.207
Female	96 (23.5)	54		
Male	312 (76.5)	48		
pN			49.640	<0.001
0	227 (55.6)	62		
1	103 (25.2)	41		
2	78 (19.1)	27		
Grade			3.605	0.132
High	169 (41.4)	45		
Intermediate	206 (50.5)	52		
Low	33 (8.1)	58		
Histology			1.685	0.194
Adenocarcinoma	189 (46.3)	52		
squamous	219 (53.7)	48		
Pleural invasion			18.903	<0.001
Yes	231 (56.6)	42		
No	177 (43.4)	60		
Vessel invasion			9.446	0.002
Yes	132 (32.4)	41		
No	276 (67.6)	54		
The 7^th^ T stage			44.450	<0.001
T1a (≤2 cm)	58 (14.2)	72		
T1b (>2 cm, ≤3 cm)	93 (22.8)	54		
T2a (>3 cm, ≤5 cm)	140 (34.3)	51		
T2b (>5 cm, ≤7 cm)	69 (16.9)	43		
T3 (>7 cm)	48 (11.8)	21		
T4	—	—		
The 8^th^ T stage			43.398	<0.001
T1a (≤1 cm)	8 (2.0)	100		
T1b (v1 cm, ≤2 cm)	50 (12.3)	68		
T1c (>2 cm, ≤3 cm)	93 (22.8)	54		
T2a (>3 cm, ≤4 cm)	93 (22.8)	52		
T2b (>4 cm, ≤5 cm)	47 (11.5)	49		
T3 (>5 cm, ≤7 cm)	69 (16.9)	43		
T4 (>7 cm)	48 (11.8)	21		

**Table 2:** Two multivariate analysis models of disease free survival according to 7^th^ edition and proposed 8^th^ edition in 408 patients with NSCLC.

Factors	HR (95% confidence interval (CI))	P value
(A) 7^th^ edition (−2 log likelihood: 2336.107; AIC value:2342.11)		
The 7^th^ T stage	1.330 (1.174–1.506)	<0.001
N stage	1.554 (1.315–1.836)	<0.001
Pleural invasion	1.395 (1.034–1.882)	0.029
(B) 8^th^ edition (−2 log likelihood: 2335.824; AIC value: 2341.82)		
The 8^th^ T stage	1.230 (1.125–1.346)	<0.001
N stage	1.569 (1.329–1.852)	<0.001
Pleural invasion	1.393 (1.033–1.878)	0.030
